# Negotiating a physically active life in tune with ageing: a grounded theory study of older persons’ experiences of participating in high-intensity interval training

**DOI:** 10.1186/s12877-024-05635-5

**Published:** 2025-01-04

**Authors:** Helena Fridberg, Maria Wiklund, Fredrik Snellman, Erik Rosendahl, Mattias Hedlund, Carl-Johan Boraxbekk, Nina Lindelöf

**Affiliations:** 1 Community Medicine and Rehabilitation, Physiotherapy, Umeå University, Umeå, Sweden; 2 Department of Social Work, Faculty of Social Sciences, Umeå University, Umeå, Sweden; 3 Institute for Clinical Medicine, Faculty of Medical and Health Sciences, University of Copenhagen, Copenhagen, Denmark; 4 Institute of Sports Medicine Copenhagen (ISMC) and Department of Neurology, Copenhagen University Hospital Bispebjerg, Copenhagen, Denmark; 5 Department of Diagnostics and Intervention, Diagnostic Radiology, and Umeå Center for Functional Brain Imaging (UFBI), Umeå University, Umeå, Sweden

**Keywords:** Older people, Physical activity, Exercise, High-intensity intervals, Qualitative research, Ageism, Stereotype embodiment, Self-efficacy

## Abstract

**Background:**

Physical activity and exercise are promoted worldwide as effective interventions for healthy ageing. Various exercise initiatives have been developed and evaluated for their efficacy and effectiveness among older populations. However, a deeper understanding of participants’ experiences with these initiatives is crucial to foster long-term activity and exercise among older persons.

**Methods:**

A constructivist grounded theory study was conducted to explore the experiences of older persons participating in a supervised group supramaximal high-intensity training (HIT) programme. Four focus groups were held, involving 28 persons aged 65 to 78. The focus groups were analysed inductively, followed by an iterative process of abstraction, abduction, and theory generation using a constant comparative method. A conceptual framework comprising three theoretical concepts—stereotype embodiment, ageist practices, and self-efficacy—was employed during the abductive phase as an analytical lens.

**Results:**

The core category of our grounded theory, *Negotiating a physically active life in tune with ageing*, encapsulates the complex processes and actions influencing older persons as they engage in physical activities in their daily lives and in relation to HIT. This core category was created from the conceptual framework and the four categories: *Grit in the moment and overall life*, *Empowered by the training group*, *Navigating one’s physically active self*, and *Committing to exercise for duty and pleasure*. Participants reported feeling invigorated by the exercise, enjoying the challenge, and valuing the group setup for its social connectedness and structure. The generated theory illustrates how participants’ engagement with physical activity and exercise is shaped by various perspectives accumulated over their lifespan. The findings provide a plausible explanation of how participation in HIT groups can challenge negative age stereotypes and ageist practices while enhancing self-efficacy for high-intensity exercise.

**Conclusions:**

Our grounded theory underscores that physical activity and exercise should be regarded as multifaceted processes, which must be considered when promoting physical activity initiatives for older persons. By considering the older person and societal norms and values, we can gather knowledge to design physical exercise interventions that are not only effective but also enjoyable and capable of transforming how individuals perceive themselves as exercising persons.

**Supplementary Information:**

The online version contains supplementary material available at 10.1186/s12877-024-05635-5.

## Background

As we grow older, biological deterioration with a decline in our bodies and minds is often highlighted as a signum of our ageing process, and one which ultimately affects our health over time [1, 2]. However, this ageing process is highly diversified and older people should be viewed as a heterogenic group with a wide range of ageing trajectories apart from chronological age that impact this process [3–5]. Healthy ageing can be defined as ‘the process of developing and maintaining the functional ability that enables well-being in older age’ [6]. Healthy ageing depends on a range of factors, such as our genetics, sex, the family we are born into, the social and physical environment we live in and our socioeconomic status [7, 8]. Hence, a multitude of determinants can be targeted to increase healthy ageing in the older population [7, 9].

Physical activity, including exercise, is a determinant that has consistently been shown over the past 30 years to be both a strong predictor and an effective intervention for healthy ageing [10, 11]. Positive effects for older persons (> 65 years) committing to physical activity are well known and include increased fitness, health, cognitive functioning, functional capacity, engagement, motivation, psychological well-being, and social inclusion [12]. Moreover, older people in Sweden who responded to a question about what makes them feel good listed being physically active as particularly important [13]. However, despite older persons accounts’ of the importance of exercising, along with a robust evidence base and national recommendations and efforts to increase physical activity and exercise for older people, less than half of Sweden’s older population meets the recommended guidelines [14]. Increased research aimed at untangling and enhancing our understanding of the driving mechanisms behind physical activity and exercise behaviour in the older population is therefore paramount. Qualitative research that focuses on the drivers of older persons, such as their experiences, needs, wishes, and aspirations regarding physical activity and exercise within their social context, is invaluable and has the potential to provide critical insights for those working to promote physical activity and exercise among the older population across society [15–18].

One important determinant for healthy ageing, apart from physical activity and exercise, is how we think, feel and act towards others and ourselves based on age, i.e., ageism [19]. A growing body of research has demonstrated that negative perceptions of old age and the ageing process are associated with unfavourable health outcomes and reduced longevity [20–22]. Moreover, there is an association between physical activity and views of ageing [23, 24]. Interventions targeting both ageism and physical activity have demonstrated that fostering more positive views of ageing is linked to higher levels of physical activity, which is also sustained over time [24–27]. Altogether, these studies point towards the importance of positive age messaging for increasing the levels of physical activity in the older population. In addition, given the high heterogeneity of the ageing population, various forms of exercise and physical activity, including modalities, intensities and doses of exercise, should be acknowledged and considered for and by each individual older person in line with their preferences and needs [28].

High-intensity interval training (HIT) is one example of a well-established exercise modality that efficiently maintains and strengthens cardiovascular function in people [29]. However, despite its physiological benefits, high-intensity intervals have been subjected to scrutiny within the research community [30, 31]. This is due to factors such as the intervals being perceived as unpleasant and challenging to adhere to, particularly for unmotivated individuals, and it is generally not considered by, nor prescribed for, older people [30–33]. To overcome these barriers, our research group has developed and evaluated a novel approach to interval training—the Umeå HIT program [34, 35]. This training programme has been adapted for older persons using controlled supramaximal six-second intervals. These short but intensive intervals of exercise are designed to activate muscles close to their maximal potential, thus promoting both leg muscle strength and aerobic capacity [34–37]. Results from the randomised controlled trial (RCT) in the Umeå HIT program, showed positive effects on physiological and biological markers while also being safe and well-tolerated by the older persons who participated in the trial [35, 38]. To extend these results with enhanced knowledge of how participants’ ageing perceptions were articulated in situ and verbally narrated when reflecting on their experiences in relation to the HIT programme, we chose to conduct a grounded theory study.

The aim of this study was to explore older persons’ experiences of participating in supervised group training in a supramaximal high-intensity training programme.

### Conceptual framework

To deepen our understanding of older persons experiences of participating in high-intensity training, we used a conceptual framework comprising three interrelated theoretical concepts: stereotype embodiment [39], ageist practices [40], and self-efficacy [41]. Stereotype embodiment theory, first posited by Becca Levy, proposes that negative ageing perceptions (stereotypes) are often unconsciously internalised from the surrounding culture throughout our life courses, utilising multiple pathways [39]. This is a process that can be sensitised with a working definition of ageist practices as ‘constitutive practices which are permeated with our experiences of the chronological, social, biological and psychological life course. We utilise age—or some adjacent terminology that signifies age—in a myriad of different ways to organise our own and other peoples’ lives…’ [40]. Stereotype embodiment and ageism can be closely linked to self-efficacy as determinants for older people’s behaviour based on their perceived, not necessarily chronologically accurate, age. Self-efficacy refers to an individual’s belief in their ability to perform specific tasks or achieve desired outcomes, and plays a crucial role in shaping behaviour, motivation and resilience throughout a person’s life course [41, 42]. The interplay between stereotype embodiment, ageist practices and self-efficacy are used in this study to conceptualise and contextualise our understanding of how older persons perceive themselves and navigate their world when they reflect on their experiences of participating in HIT.

## Methods

### Design

A constructivist grounded theory (GT) approach, as proposed by Charmaz [43], was used to generate a contextualised understanding of older persons’ experiences of participating in the HIT programme. It resonates with our stance that reality is a subjective construction created between the researchers and participants, whereby we as researchers ‘offer an imaginative theoretical interpretation that makes sense of the studied phenomenon’ [43]. As utilised in this study, the methodology of constructivist GT is open to using theoretical frameworks and sensitising concepts along with inductive analyses, resulting in the analysis of data through an iterative and abductive process [43]. The theoretical concepts of stereotype embodiment, ageist practices and self-efficacy emerged as useful to interpreting and explaining our observed data during the later stages of our analysis and theory generation.

### Setting

This study took place in northern Sweden in the municipality of Umeå, which has 130 000 inhabitants spread across urban and rural areas. Umeå is a university town with a mean age among inhabitants of 39, which is slightly younger than the entire country of Sweden (~ 2 years). The area has large seasonal changes, including winters with plenty of snow and summers where the sun is up more than 20 h a day. Swedish people are generally (along with the other Nordic countries) more physically active than people living in other countries in Europe. This not only concerns exercising by doing sports but also in relation to other forms of physical activity such as cycling or gardening [14].

### Study context

**Fig. 1:**
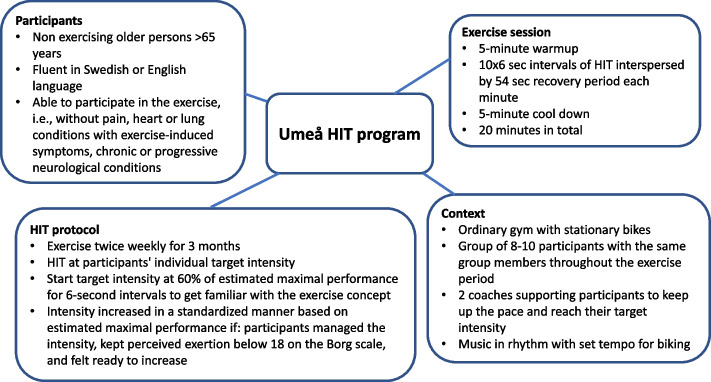
The Umeå HIT program, describing the participants, exercise sessions, context, and HIT protocol (for full details of the programme, see Simonsson et al. [35])

This study is part of a larger project, the Umeå HIT study, aimed at evaluating the applicability and effects of supramaximal HIT compared to moderate-intensity training among non-exercising older persons [34, 35]. Participants in this study were recruited from the group who had been randomised to take part in supramaximal HIT [35] (Fig. 1). The RCT has been registered at https://clinicaltrials.gov/study/NCT03765385.

### Participants

All participants from the high-intensity exercise groups were approached by e-mail and asked to participate in focus groups (FGs). A total of 28 persons out of 34 from the four supramaximal HIT groups participated in the FGs. They were evenly represented by men and women and their ages ranged from 65 to 78 years. The majority lived with a partner and had a university degree. Approximately three-quarters of the participants had engaged in regular exercise at some point in their lives. They attended between 11 and 25 HIT sessions out of 25 (median 23) [35].

### Data collection

FGs were conducted between April 2019 and January 2020, directly after each HIT group’s last exercise session, in the same groups as during the training, and in a secluded room at the training facility. Each focus group (*n* = 4) contained three to ten participants, including both women and men, and lasted from 57 to 69 min. At the time of the interviews, participants had not received any information about individual or overall effects shown from taking part in the HIT training.

**Fig. 2:**
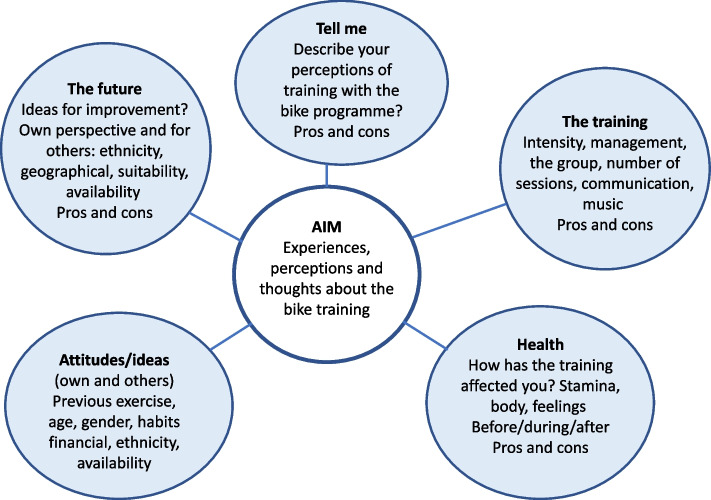
Themes in the semi-structured interview guide used with older persons who shared their experiences of participating in the Umeå HIT study

A semi-structured interview guide was used with open-ended questions and suggested themes feeding from one interview to another. The participants were given information about the aim of the FGs and the researchers’ role. During the interviews, the last author (NL) acted as moderator with assistance from the second author (MW). MW had never met any of the participants prior to the interviews while NL had met two of the participants during the screening process for the RCT. Both authors are skilled interviewers and identify as women. An introductory question was posed in which the participants were asked to ‘please describe your experiences of training with the bike programme’. The moderator let the participants discuss freely without much interruption but made sure they touched on the major themes in the interview guide (Fig. 2).

Discussions from the focus groups were audio-recorded and transcribed verbatim by two bachelor students (*n* = 3) and a professional transcriber (*n* = 1) not involved in the study. Directly after each interview, memos were written in which the researchers briefly summarised and compared their initial understandings of the centralities and details of the FGs, such as content, group process, individual contributions, and emerging themes or patterns.

### Data analysis

The analysis was informed by constructivist GT [43] and conducted in two phases, starting with an inductive process, which was followed by an abductive process, i.e., altering between empirically generated analyses and theoretically informed understanding. Open coding was initially conducted line by line to capture the meaning of what was said. Open codes were then evaluated, sorted and synthesised into focused codes containing similar actions. These focused codes were further clustered and abstracted into the generation of subcategories and categories. During these stages of the inductive process, data were constantly compared and discussed among the research group, leading to refinements and successive synthesising of data. Subcategories were constructed to outline the dimensions within each category and to make the analyses more accessible and transparent for the larger research group. At this stage, a tentative core category was formulated. Thereafter followed an iterative process of abstraction, abduction and theory generation. During this phase, the theoretical concepts of ageist practices, stereotype embodiment and self-efficacy were incorporated to guide our interpretation and theory generation. Ageist practices and stereotype embodiment were initially identified as useful for understanding our empirical data. In the later stages of abduction, the theoretical concept of self-efficacy emerged as a valuable addition in the conceptual framework. Memos were used throughout the data analysis and tentative models were created, discarded, recreated, and refined throughout this process.

The coding process was conducted by the first author (HF) in close collaboration with NL and MW. In a process of triangulation between researchers, the authors (HF, NL and MW) held regular meetings throughout the analysis process to discuss the emergent codes, categories, and the theoretical model. Reviews of the analyses in relation to the theoretical concepts, i.e., the abductive process, were held at regular intervals with FS. Later in the process, the focused codes and categories, along with the preliminary theoretical model, were discussed with the whole research group, which led to further changes and refinements. The analysis was conducted in the software NVivo version 14.

### Reflexivity

While all authors take an epistemological stance of regarding the study inquiry and results as a process of co-creating knowledge between researchers and study participants, we also acknowledge the importance of being transparent and reflexive regarding our preconceptions about the studied phenomena.

The authors come from a variety of backgrounds, disciplines, and research areas, and are familiar with a variety of different research methodologies. Before and during data collection, analysis, and drafting of the results and the manuscript, the authors (NL, MW and HF) used ongoing reflective memos and all authors shared their preconceived notions of ageing, health and exercise [43].

## Results

**Fig. 3:**
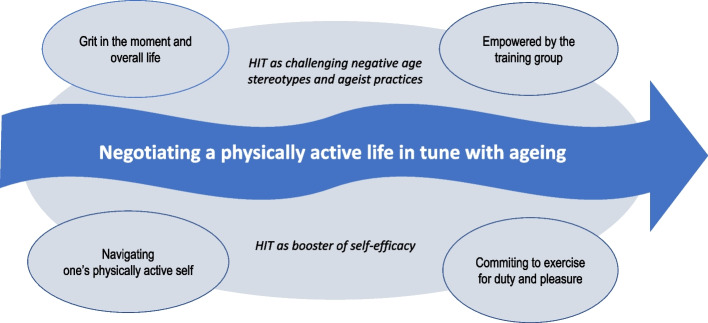
A GT model of older persons’ experiences of participating in HIT

We have constructed a GT that revolves around the core category *Negotiating a physically active life in tune with ageing.* This refers to participants’ experiences of engaging in HIT interwoven with reflections on the uncertainties of ageing and strategies for maintaining a physically active lifestyle. The core category captures an ongoing process of negotiating healthy and active ageing intertwined with key processes of being empowered and gaining more grit when participating in the HIT exercise group (Fig. 3).

Our GT was informed by the conceptual framework, i.e., stereotype embodiment, ageist practices and self-efficacy. The conceptual framework permeates all four categories and the core category, acting as a plausible explanation of how participation in HIT groups challenges negative age stereotypes and ageist practices, and boosts self-efficacy for high-intensity exercise.

Moreover, age is a central part of the core process as an ever-present factor and represents the uncertainty of ageing that participants described in relation to, for example, physical activity and exercise behaviours. Age-related limitations were described as a reality that needs to be considered and accommodated for, or as an imaginary threat lurking in the outskirts of participants’ minds, described as something that may come to affect them in the near or far future.

Our GT captures participants’ individual experiences and collective negotiations, which are also related to norms and values in the larger society. Participants’ views of themselves, including their age and their previous experiences of exercising, shaped how they regarded physical activity, exercise, and the HIT programme itself. Thus, their lived experiences, from the past to the present, along with thoughts about the future, unfolded a variety of processes that reciprocally shaped and affected them when they participated in the HIT groups.

The core category was created from four distinct yet interconnected categories: *Grit in the moment and overall life, Empowered by the training group, Navigating one’s physically active self,* and *Committing to exercise for duty and pleasure*. Each category represents a variety of actions and processes derived from our empirical data. The data analysis process is outlined in Additional file 1. The categories are described in detail below, along with examples of interspersed references to the conceptual framework to elucidate the interconnections between empirical data and the conceptual framework. The interconnections are further elaborated in the discussion section.

Direct quotes in the text are denoted M for male and F for female participants along with each focus group number.

### Grit in the moment and overall life

This category was created based on participants’ experiences of successively gaining ‘grit’ in relation to training in the specific HIT programme, but also on how this feeling of grit carried over into their everyday life. Our interpretation of grit is, for instance, expressed in terms of perceived ‘capability, stamina, and motivation’, which also corroborates the concept of self-efficacy. Depending on previous and present experiences of physical activity and exercise, participants approached the HIT in various ways.

#### Vitalised by challenging oneself

The HIT programme was considered a way to break an inactive lifestyle while also being fun and easy to commit to. It served as a reminder that older persons can push hard through intervals. For others, the HIT programme brought a welcome surprise and insight into their capability to do interval training, as a ‘fun’ way of exercising. This newfound experience of challenge and feeling vitalised led them to reconstruct and negotiate how they viewed themselves as exercising individuals with capabilities far beyond what they had thought possible. These positive experiences can be interpreted to challenge negative age stereotypes and ageist practices and to lead to a boosting of efficacy beliefs through mastering of HIT.

While many participants had been accustomed to exercising in the past, narratives of a decline in strenuous exercise as one became older were told, and these participants declared that they had truly enjoyed ‘pushing hard’ on the bikes during the intervals.

*-Well yes, it was fun to [exercise]...that’s what you did in your youth, you did some sports. And then [in your youth] it was like you almost spit blood in the end. But I found that this was great, to sort of exert yourself a little and feel that you really had to push yourself. Yes, really great.* (M)

*-Yeah, and not just plod on, in a kind of normal walking pace. *(M, FG1)

The intense intervals were further discussed by two participants:

*-I’ve almost been looking forward to these six seconds.* (M)

*-When you get to push yourself.* (F)

*-Yes, that’s right, that you get to push yourself, yeah. And then... go down to zero and then...you charge up again. Damn it, you think, now I’m going to get through this. So that...I think that was really good.* (M, FG1)

Feelings of happiness, satisfaction, and being invigorated both during and after HIT were described. Some even would have liked to have longer and harder training sessions, especially in the beginning of the training period, as they, despite the intervals, ‘didn’t get sweaty or tired’ and didn’t get any muscle soreness in between sessions. A different perspective was given by participants with less experience of hard exercise. They described how the setup helped them understand how they should feel during the intervals, and how they enjoyed challenging and pushing themselves more than they were used to.

*-Yeah, for me it was perfect because I had entered a bit of a lazy stage in life. When the sofa felt nice and comfortable, but always with a guilty conscious about not doing anything. This form of short and intense* [exercise] *using such a small part of the day as possible suits me perfectly. I think it’s great.* (F, FG4)

#### Harvesting the benefits of exercising

Perceptions of the benefits of exercising with HIT varied across a wide spectrum from no distinct perceived effects to experiences of finding it easier to cycle and walk uphill, getting stronger leg muscles, and experiencing easier breathing during exercise or lesser feelings of exertion despite higher resistance during HIT. Having positive experiences from exerting oneself and increasing one’s pulse during the training was described as carrying over to other activities and situations in which the participants pushed themselves harder than usual and tried to mimic an interval by, for example, walking or biking faster uphill. These narratives elucidate a generalisation of increased efficacy beliefs for exercising through positive interpretations of physiological states, e.g. increased pulse, as something desirable and positive to strive for.

Other perceived benefits from HIT included improved feelings of well-being and sleep, as well as needing fewer hours of sleep but still feeling more rested. There were also experiences of being more positive and alert, with more drive and motivation to do things in general.

*-Well, you have a little more energy and become more motivated to do different things because you feel like you have the energy and stamina to do it. It can be like small things around the house, you know, but now I’m doing it. I’m not just sitting there in front of the telly anymore. I started painting the house a few days ago, maybe I hadn’t... I probably would have thought it through a bit more before, how to do it, but now I just got started, boom, full steam ahead.* (M, FG1)

While some individuals were resolute in their beliefs about the valuable effects of HIT, others expressed hesitance regarding any perceived benefits. The sceptics did not believe that HIT could yield any significant results, as they did not get tired or perspire much during the sessions. A prevailing belief was that effective exercise must result in participants feeling ‘tired and sweaty’. Consequently, the training was perceived as too undemanding to yield any substantial effect. However, those harbouring doubts conveyed that if the HIT programme demonstrated favourable outcomes, they would recommend it to everyone they knew, as it was perceived as such an easy way to exercise.

### Empowered by the training group

This category is based on participants’ experiences of being empowered by exercising in a group setting. Together with their peers, they developed a sense of capability and motivation to persist with exercise as well as drive to explore alternative exercise modalities and training facilities while preparing for ongoing exercise beyond the completion of the training project. These results point at increased self-efficacy for HIT and a generalisation of increased self-efficacy for other kinds of exercise behaviours, as well as a challenging of negative age stereotypes and ageist practises.

#### Acknowledging the merits of exercising in a group

An abundance of valuable benefits connected to exercising in a group were experienced. Having set times and days for exercising with the same individuals gave a structure to their week that facilitated devotion to exercise, as well as a positive feeling of needing to attend each session as the others in the group would otherwise notice that they were absent. They perceived that the group helped them continue with their training and did not think they would have done that had they exercised on their own.

*-I think it becomes automatic if you are a group, you have to go there. If you’re on your own, ‘I couldn’t be bothered today—I’ll do it tomorrow’. But when you’re in a group, you have a set time and you’re part of that group. Then you must go.* (F)

*-I think that’s important too, having a time and that you have planned for it. If you’re doing it on your own, then it’s a little bit, the lazy side takes over.* (M, FG4)

During each session, the group and the coaches helped them to exert themselves more than if they had been exercising on their own. The chance of reaching their set goals for each session was greatly improved by seeing the other group members pushing themselves. Moreover, they started to create bonds and social connections with each other in the group, which was likened to ‘therapy’. One group described how they started to meet up for a bit of a chat before each training session, and another group decided to have coffee once a week after their session. Thus, participating in the training not only involved being active and exercising but was also found to develop an important social connectedness between participants.

*-I think it’s positive. I can come and have a fairly low level of... mentally ... and find things quite challenging, both this and that. But... the group has given me more energy. And afterwards I’ve felt that my mood has improved. At least a bit, sometimes even more. So for me it’s important to have this positive group.* (F)

-*We’ve usually met up a bit before, and then we’ve been sitting there chatting about everything and nothing. Life and death and, everything.* (F, FG4)

Another special connectedness involved the coaches who ran the exercise sessions. Coaches were seen as a very important part in the overall HIT experience and participants felt ‘taken care of’ and ‘seen’ by the coaches, which was described as a thoroughly positive and welcoming experience.

#### Preparing for continued exercise without the group

This category entails discussions about different actions that participants took to continue exercising regularly on their own, without the HIT group. Individuals who had been used to exercising on a regular basis previously seemed to be more inclined to find ways to continue and plan for ways to exercise when the project was coming to an end. The positive effects they had experienced of the HIT were expressed as a drive to continue exercising. Their drive led to varied actions, such as looking for gyms that offered similar bike training in groups, buying new or dusting off their own exercise bikes, which they intended to use at home, rallying fellow residents in an apartment building to persuade the owner to invest in gym equipment, and exploring alternative exercise options in nearby gyms. Some participants intended to commit to outdoor activities like walking and cycling. An intention to continue with interval training was discussed, but finding the appropriate resistance levels aligned with the HIT programme was perceived as somewhat challenging to do on one’s own. Ultimately, some declared that they were going to rely on their ‘gut feeling’, pushing hard during intervals, resting, and then repeating the process. While a range of innovative ways to maintain exercise routines were talked about, there were also participants who exhibited hesitancy and uncertainty about exercising independently.

*-But then, I would really like to continue, I have to, I want to continue with this, but how? What if I miss this* [high-intensity interval training] *as well, will I fail and drop this?* (F, FG3)

Concerns about missing the group setup and doubts regarding their ability to perform intervals independently were expressed. Thus, while some were hesitant about their ability to continue to exercise on their own, others questioned their capacity to sustain high-intensity intervals or opted directly for a return to their prior light activities without intervals. Such narratives corroborate self-efficacy theory, illustrating how self-efficacy for HIT among these participants is directly related to performing the specific behaviour, the high-intensity intervals, in the HIT group and has not generalised to other settings.

### Navigating one’s physically active self

This category refers to participants’ navigating of their physically active self by engaging in HIT and provides a broader context to their experiences of staying active. Discussions in FGs flowed naturally and contained jokes and laughter about the merits of ageing mixed with serious narratives about illness and death and the hardships associated with advancing age. Participants described ‘healthy ageing’ in relation to staying active, as an ongoing process affected by factors such as their physical health and perceived ability to exercise, relatives and friends, the larger society, and the crucial transformation that happened when they entered retirement.

#### Relating to one’s own active ageing in social contexts

The nature of ageing in relation to being active was described as a challenging and an ongoing uncertain process in which participants thought that being old and healthy should not be taken for granted. Accepting one’s ageing was an ambiguous process in which feelings of being grateful for fairly good health and doing the best with what you have were juxtaposed with descriptions of feeling sad about not being able to continue with activities that one had previously enjoyed. This ambiguity was further elaborated in relation to participants’ chronological age, where some said that they had a hard time identifying as an older person, indicating a rejection of negative age stereotypes to their self-image.

Thus, ageing was often described in relation to other individuals and the society. Seeing and experiencing other people getting older shaped the participants’ own identities and perceptions of ageing.

*-Well, we are actually older, and it’s…* (M)

*-And we should be happy that we’ve come this far.* (F)

*-And there’s arthritis, and there’s heart problems and blood pressure, and things like that… and then there’s those who get cancer also, but it’s always these problems that you read about and hear when talking to people.* (M)

*-I know people my age who have already been affected by dementia. So of course… you never know what will happen to you.* (M, FG1)

Reaching old age was not described as a strong wish or priority. Instead, they acknowledged a hope and preference to have health and well-being while they were living.

Challenges related to accepting one’s ageing and belonging to the older generation came with a sense of ambivalence and tension, where participants portrayed themselves through an ‘us and them’ narrative. Thus, they described themselves as disparate to older and younger generations alike. This ambivalence appeared, for example, in portrayals of themselves as being far more physically active nowadays and ‘not as old’ as previous generations of older persons. These narratives suggest that some participants had endorsed negative views, age stereotypes, about earlier generations of older persons but rejected these stereotypes when considering their own self-image.

In contrast, younger generations were depicted to differ in other ways. Participants viewed themselves as more mature than younger generations, without a need to compete or compare their appearance or physique with others. To avoid ‘feeling like outcasts’, some described how they had previously exercised during daytime hours, deliberately avoiding gym sessions with younger individuals.

*-Some* [younger] *people really feel the need to show off, and they can barely use any equipment until they make sure someone is noticing how skilled they are. It’s so foreign to me that I feel like saying ‘get a life’. Is that what life is about? So, you just have to ignore them, do your workout, and then leave. But of course, they also look at us, naturally, so you just have to close your eyes and keep your thoughts to yourself.* (M)

*-Go during the day*. (F)

*-Yes, you should go during the day because they [the younger generations] are often there in the morning and evening*. (F)

*-In the evenings, all dressed up and made up, it’s so fancy. You start wondering if you yourself came from the countryside*. (F)

*-No, you should go during the day; then you can be whoever you want.* (F, FG4)

Thus, having difficulties in identifying with other generations led to a desire to exercise with people they felt more closely connected to—their age group.

#### Trying to stay active in the transformation to pensioner

Becoming a pensioner was discussed in depth and was closely related to discussions about ageing, activity and exercise. It was depicted as a major life event in relation to ageing and forced participants to transition into finding and constructing a new identity. This transition into retirement can be related to age stereotypes and functioned as an arbitrary passage into the identification as ‘old’ for some of the participants. Transitioning to life as a pensioner meant that they had to adjust to a new lifestyle outside their work environment, and try to find new priorities and meaning in their lives. Finding this new identity was described as a life-changing process invoking ambivalent perceptions of the advantages and disadvantages of staying in the workforce. Becoming a pensioner was portrayed as entailing challenges related to staying active and exercising. Whilst in the workforce, staying active was facilitated by several processes, such as biking or walking to work each day. Moreover, participants described how they used to exercise with colleagues on a regular basis and that exercising was encouraged by their employer through subsidised gym cards and time off for training during working hours. When these supporting actions disappeared as they left the workforce and became pensioners, the participants experienced difficulties finding their own routines and drive to stay active, as expressed in rich descriptions:

*-Now you have to do everything yourself. Even if you have all the time in the world, you still have to manage all the projects yourself. I think that’s a real difference. When you worked, you got so much for free in a way. Even though you were working, it felt like you still did more.* (F)

*-That’s exactly how it is, and the more you do, the more motivation you get to do more, because when you become inactive, it’s like…* (M)

*-Then nothing happens. …then it becomes a vicious circle.* (F)

*-That’s how it is.* (F, FG3)

A sense of becoming insignificant and left alone upon becoming a pensioner was discussed.

Moreover, apart from needing one’s own drive to exercise and stay active, the participants also acknowledged how people around them somehow treated them differently due to their increasing age and physical capability.

*-My sons kind of complain… they say I’m old, ‘No, you shouldn’t stand at the top of the ladder and paint, no, you shouldn’t do this and that’.* (M)

*-It’s just concern* (M).

*-But common, I’ve done that before. ‘Yes, but now you have to consider that you’re older, your balance is worse’, and yada yada. Have I suddenly been declared incompetent just because I’m a pensioner?* (M, FG2)

Perceptions of being treated differently were also recounted in relation to the HIT programme, where participants thought that the resistance was too low in the beginning of the exercise period. They were unaware of the HIT setup, which involved beginning their training period at a lower resistance level than they were capable of tolerating. This deliberate approach was part of the protocol designed to help participants become comfortable and familiar with the high-intensity intervals. However, this was mistakenly interpreted as a product of their age, with the assumption that the coaches were overly concerned about their health before allowing them to increase the resistance on their bikes. Interpretations such as these suggest that participants identified as members of the older population while questioning potential ageist practices and negative age stereotypes manifested by relatives and the coaches.

### Committing to exercise for duty and pleasure

The process and drive to exercise were discussed from several perspectives, resulting in a category with various connotations shaped from participants’ experience of exercise, their current individual life situation and health status, as well as external drivers from family and friends, or society. These discussions can be directly related to stereotype embodiment, ageist practices and self-efficacy theory. Theoretical underpinnings in our conceptual framework stipulate that the images that we construct of others, and ultimately of ourselves, based on age operate in two directions—longitudinally throughout our lives and top-down from the surrounding society to the individual. Participants acknowledged that various factors could act either as supportive or as barriers to committing to exercising. Moreover, while some committed to exercise out of sheer pleasure and joy, others perceived it as a duty they had to uphold to maintain their health.

#### Finding own drive to exercise

One reason to commit to exercise was the desire for health and well-being and being able to live an active lifestyle for as long as possible. Maintaining health by exercising was also perceived as a way of fulfilling one’s civic duty as a responsible citizen. This drive to exercise was linked to the desire to delay the need for support from relatives and society, and to avoid becoming a burden on others. Thus, a strong wish to stay autonomous in increasing age was important and contributed to participants committing to exercise.

*-Well, I guess that the motive is to have the best possible old age and avoid being taken care of when you’re 80.* (M)

*-That one should be able to feel good for as long as possible.* (F, FG1)

Other drivers for committing to exercise were to have a purpose, something to do to fill the days with, or a way of breaking a sedentary lifestyle due to previous ill health or, as some described, ‘laziness’. Another major reason to commit to exercise was that the action of exercising was regarded as fun and pleasurable in its own right and led to feelings of increased well-being.

Finding one’s own drive to exercise also entails claims about participants’ self-images, not only based on their age, in relation to their experience of exercise, i.e., how they viewed themselves as exercising persons. Their self-image seemed to be based on experiences in the past as well as the present, which is in line with teachings on self-efficacy stipulating that former experiences of mastering, e.g., physical exercise behaviours will strengthen a person’s efficacy beliefs and chances of executing this behaviour in the future.

#### Acknowledging social support and availability to exercise

This subcategory was shaped from descriptions outlining support from family and friends and the larger society, e.g., the municipal and local training facilities as well as the nature and environments in the participants’ surroundings, which were acknowledged as helpful or hindering for exercise behaviours.

Relatives were sometimes both a major influence and driver for participants to commit to exercise. Many had spouses that cheered them on to exercise and they were also seen as great companions during physical activities such as going for walks in the forest or riding a bicycle. Getting verbal support from others and seeing other older people exercising, such as spouses and friends, can be seen to strengthen self-efficacy beliefs through modelling and verbal persuasion behaviours from important others. Descriptions were made of receiving gym passes for Christmas, and how their children were supportive of their wish to exercise.

*-I have received applause from children and the whole family, all relatives, ‘Mary has finally started to move’.* (F, FG4)

Knowledge of the importance and merits of exercising were often discussed between participants in the FGs, creating a sense of being responsible for and having a responsibility for one’s own health. Recollected news reports and government campaigns were brought up in discussions. However, having this knowledge did not necessarily carry over to regular exercise behaviours. Instead, their commitment to exercising was deeply affected by their opportunity to stay active. High costs associated with joining a training facility, long distances or difficulties in finding transportation to a training facility, troubles finding suitable exercise classes, and set gym times for older persons with subsidised memberships were described as barriers to exercising.

*-Yes, I think that they could be more generous towards us over 65 and that it* [set gym times] *shouldn’t only apply from 7* [am] *to 1* [pm]. *Take myself as an example. I’ve only been able to do yoga and that class is only given at, well, eight o’clock in the evening at XX* [a local training facility]. (F, FG3)

To beat the financial barrier for older persons to exercise, participants suggested that training facilities should be free for all people in the older population. This was narrated from a win–win perspective where older people would reap individual health gains from being more active and the government and health-care sector would save resources in the long run due to a healthier older population.

Physical activities and exercise were also discussed in relation to seasonal changes. Depending on the season, participants described how they, for example, cross-country ski and shovel snow in the winter, and cycle, work in the garden, and go for walks in the summer. Moreover, seasonal changes also affected their ability to get to training facilities.

*-Well, I’ve lived close to the gym, so it’s been ok. Because it can’t be too far away, if you have to start the car and its icy and no, then it’s easy to back away. It* [the training facility] *has to be easy to get to.* (F)

*-I think so too. Close, and easy to get to.* (F, FG4)

## Discussion

Our main results, the generated GT with the core category *Negotiating a physically active life in tune with ageing*, depicts an elaborate web of interactive processes and actions that come into play when older people negotiate physical activity and exercise in relation to HIT as well as in their daily lives. Our GT encompasses processes from a broad societal context beyond the HIT setting, incorporating societal norms and values. The conceptual framework has enabled us to extend our interpretations with the perspectives of ageist practices, stereotype embodiment and self-efficacy theory.

The categories *Grit in the moment* and *Empowered by the training group* are directly related to experiences of participating in the HIT. The exercise programme was described as new, fun, pleasurable and easy to commit to. These participant experiences stand in contrast to research that casts HIT as an uncomfortable mode of exercise, potentially discouraging rather than motivating sustained engagement [30, 31, 33]. This disparity may stem from the notably brief six-second intervals utilised in the HIT programme, which presented a challenge to participants without necessitating ‘all-out’ efforts commonly associated with unpleasant experiences. Our findings suggest that exercising in bursts of controlled six-second intervals can foster positive affective responses, potentially enabling older individuals to maintain this exercise routine over the long term, particularly when supervised and conducted within a group setting.

We introduced the concept of self-efficacy into our conceptual framework when our in-depth analyses elucidated a connection between our emerging results and self-efficacy theory [41]. We suggest that the HIT programme has a potential to enhance older people’s efficacy beliefs regarding exercise by incorporating several aspects known to influence a person’s estimation of self-efficacy [41]. The most influential source to altering a person’s efficacy belief is by way of experiencing and mastering a specific task [41]. This was achieved in the HIT programme by having participants successively raise their resistance each time they reached a set target. Thus, they managed to do the intervals at the prescribed pace and dose and described a successive feeling of managing the exercise setup [41]. Secondly, they were training with persons in the same age group, creating a role-modelling effect on each other, i.e., adopting a mindset of ‘if that person can do it, so can I’ [44]. Third, the coaches were regarded as trustworthy and a great source of support in each exercise session by checking and encouraging participants to increase the resistance when they had reached a set target, thus increasing efficacy beliefs through verbal persuasion and feedback from important others [41]. Lastly, participants went to the exercise sessions challenging previous ideas about their physical ability and learning how to interpret their physiological and mental responses when they carried out the intervals. This relates to the last source of altering a person’s efficacy beliefs, i.e., learning how to interpret somatic and affective states as natural and desirable reactions [41]. Moreover, we learned from many of the participants that efficacy beliefs also carried over to other types of behaviours in their everyday lives. Narratives described striving to find other activities where they could mimic intervals and increase their pulse, as well as a sense of having more drive to do all sorts of things around and outside of their home environment.

The results from our GT study did not align with the survey responses to the exercise self-efficacy scale used in the RCT, which showed no significant changes in exercise-related self-efficacy over time [38]. A plausible explanation for the discrepancy between participants’ survey responses and their discussions in FGs could be that, while Bandura strongly recommends using measures of self-efficacy specifically targeting the intended behaviour to achieve measurements sensitive enough to capture change over time [41], the exercise self-efficacy scale employed in the RCT does not specifically capture self-efficacy for supramaximal HIT [45]. A multitude of studies affirm that self-efficacy is a strong determinant and correlate for physical activity behaviours [41, 46, 47]. Efficacy belief has also been found to act as a mediator between ageism and health [21] and between self-perceptions of ageing, physical function and exercise [48, 49]. Thus, ageism can undermine an ageing person’s self-efficacy by reinforcing negative stereotypes about ageing. When we view older people—our future selves—as less capable, such beliefs may be internalised. This can lead to doubts about our abilities and influence us to act accordingly, ultimately affecting our physical, cognitive and emotional well-being [39].

Exercising with HIT was enabled by the group setting and the coaches and contributed strongly to sustaining exercise behaviours and a sense of purpose and social connectedness among participants. Similar results have been shown in other studies where older people who exercise together in group settings report perceived benefits apart from improved physical health [15, 18, 50, 51]. These benefits include a greater sense of purpose and increased self-belief [15], increased exercise enjoyment and adherence [52] and also emphasise the importance of fostering social interaction among participants [18, 50]. These results show that some persons in the older population prefer engagement in group exercise settings. The social connectedness and support fostered among group members is perceived as essential for the long-term maintenance of exercise and activity behaviours [18, 52].

When participants reflected on their experiences of participating in HIT, their discussions expanded to include rich narratives about perceptions of ageing related to physical activity in general. This contextualisation therefore led us to broaden our GT to include these narratives as well. The categories *Navigating one’s physically active self* and *Committing to exercise for duty and pleasure* contain older persons’ perceptions on ageing in relation to being physically active, and various drivers for activity behaviours in their everyday lives. These categories highlight participants’ awareness of their age and in some cases their experiences of physical and functional decline, which they try to negotiate and incorporate with their life situation and physical activity and exercise behaviours. Consequently, age and ageing as physiological and social processes are integral to understanding our generated theory. The approach helps to elucidate how these processes are not tied exclusively to the individual and their current experiences of HIT but rather concern how older people negotiate exercise and stay active from many perspectives. These perspectives are varied and underscore the heterogeneity in the older population, including past experiences of being physically active; perceptions about ageing and living with an ageing body; their wishes, aspirations and needs; support and input from important others; and influences from the society and the environment in which they live in. Our broadened GT shares many similarities with ecological models and other studies exploring older persons' exercise experiences across a range of settings, exercise programs and modalities [46, 53–56]. These similarities support the notion of viewing physical activity and exercise for older persons from a broad and comprehensive systems perspective, encompassing individual, environmental, and policy levels [57]. Our findings and previous research on exercise and activity levels in the older population, underscores the complexity within this field and the need to collaborate across a range of disciplines to gain a comprehensive understanding of what it takes to support the ageing population in increasing their physical activity levels [57–61].

We identified traces in the empirical data pointing us toward ageist practices and stereotype embodiment that cut across all our constructed categories [39, 40]. Participants recalled early memories of (i) engaging in sports and (ii) pushing themselves physically, which they had stopped doing as they aged, and discussed experiences of being viewed as old by others, for example at the gym. These two snapshot examples can conjointly be understood as constitutive practices of ageism, signifying how age is utilised in different ways to organise and understand our own life choices as well as how others are involved in that construction of reality [40]. In short, the constitutive process uncovers the reciprocal interplay between societal structures and individual choice and how ageist practices are renegotiated in society [40]. Individual choice is influenced by societal structures, such as unspoken rules of how to behave and act at a certain age, including dress codes at the gym, and whether to engage and push oneself in physical activities. This constitutive ageist practice between societal structure and individual choice is relevant for exercise behaviours like HIT. It may affect older people’s decisions to refrain from participating in vigorous and challenging exercises due to socially constructed information suggesting that such activities are not age-appropriate. Additionally, this perception may be further cemented by exercise facilities and fitness coaches if they are overly protective toward older people, offering mainly ‘light’ exercise options for people of advancing age.

Our two examples above—about pushing oneself and going to the gym—also corroborate Levy’s reasoning on stereotype embodiment [39]. The participant who spoke about pushing oneself when doing sports earlier in life recollects that memory from a specific point in time during their lifespan, suggesting an internalised subjective image about when it is appropriate to engage in HIT. It appears as if this person was surprised to experience the same type of positive feelings when engaging in HIT at an older chronological age. Following another of Levy’s assumptions of stereotype embodiment, a suppression of self-image might have led the participant to believe that they were no longer capable of participating in HIT-like exercise. Interestingly, this individual made a new discovery that challenges prevailing societal expectations, in that HIT is not exclusively reserved for younger individuals. We therefore propose that exercising with HIT to some degree challenges negative age stereotypes by reminding older people of the capacity that they still have to keep to an exercise routine and push themselves through the prescribed intervals.

The stories from the focus groups of avoiding the gym at a certain age, or during specific times of the day to steer clear of exercising alongside younger individuals, aligns with Levy’s assumption that stereotypes become more pronounced as we begin to identify as part of the older generation. By avoiding the gym at specific times during the day, the participants avoided feeling like ‘outcasts’. This serves as a symbolic example of situations of what we here label as a ‘negative age-gaze’ that leads to an identification of being old. This process involves life-course adjustments due to age stereotypes and seems to impact how individuals view themselves. The challenge lies in manoeuvring the seemingly innocuous repetition of being labelled as old, which may lead individuals to internalise these ideas and believe they are no longer capable of performing certain activities.

We acknowledge the heterogeneity of the older population and the need to understand that there is not a ‘one size fits all’ exercise regime that can be promoted across our society. Instead, we propose that HIT is one way to exercise, amongst others, which gives rise to a range of positive benefits (apart from physical effects) not often outlined in today’s physical activity narratives. Perceived benefits, such as feeling invigorated by pushing oneself in a similar manner as a younger version of oneself, having fun and enjoying the exercise, successively gaining grit for other everyday activities, and feeling a connectedness with other participants, are examples of such narratives, which can be regarded as motivating factors or drivers for exercise in both the short and long run. We suggest that these and other drivers for exercise in the older population should be accumulated and integrated with evidence from physical intervention initiatives with the aim of tapping into the desires and goals that are important to the individual older person.

### Limitations and strengths

Our results must be regarded in light of the recruited participants who applied on their own initiative to be part of the RCT. At the time of application, they knew that the project involved exercising with other people aged 65 and over in a group setting and getting a health check prior to being admitted to the project. By recruiting people aged 65 and over, we contribute to the constitutive practice of constructing a group of ‘older’ people based on their age alone. This was done for convenience and an alternative could perhaps have been to recruit people who had entered retirement regardless of their chronological age. Moreover, people from our population were fairly homogenic. Older persons born outside of Europe and with lower educational levels would perhaps describe alternative experiences of participating in the HIT programme, which is something we need to consider in future studies. At the time of the focus groups, participants had not been given any results from the RCT they had been part of. We believe that this is a strength as it decreased the likelihood of bias when participants were discussing the merits and effects of committing to the HIT programme.

Furthermore, we consider the data analysis by an interdisciplinary research group with different research traditions to strengthen the credibility of our study findings. We were able to merge the researchers’ knowledge of the specific setting and the RCT with the broad expertise on the topic, methodology used, and familiarity with the theoretical conceptual framework. Triangulation through the authors’ different backgrounds was also regarded as an important part in tracking reflexivity during the research process. To enhance originality and resonance, we introduced the conceptual framework into our study, extending our interpretations beyond empirical data by incorporating additional theoretical concepts. The conceptual framework was not defined a priori but emerged through a collaborative process of discussions among the researchers during the course of our analysis and theory generation. We acknowledge that there are other related theoretical perspectives that could have been employed in our conceptual framework, such as age-based stereotype threat [62], self-perceptions of ageing [63, 64], physical self-worth [65] and intrinsic and extrinsic motivation [66, 67]. We recognise that the theoretical standpoints we adopt are invariably influenced by the researchers’ experiences and knowledge of the research topic [43, 68].

Our goal has been to create a GT that makes sense to the reader while also generating new ideas relevant for future studies and applicable to people’s everyday lives—whether they are policymakers, professionals in exercise settings, researchers, or older people.

## Conclusion

Our GT signposts the importance of abstaining from regarding physical activity in the older population as a single entity that happens in a vacuum separate from our daily lives with societal norms and values. On the contrary, it points to physical activity and exercise behaviours as a lifelong accumulation of experiences and habits shaped by the context we have lived and live in. In addition, as each and every one of us is a member of our society, we are integral to this top-down and longitudinal ageing process. This underscores the significance of raising awareness about ageist practices and age stereotypes, as they can impact us both positively and negatively—in the present and in the future. We propose that HIT has the potential to challenge negative age stereotypes and ageist practices, and boost self-efficacy by supporting older people to exercise in a manner that they have not done for a long time or ever in their lives. By looking at the individual and beyond, we can continue to gain valuable understanding of what it takes to inspire and support the ageing population to be physically active, with the goal of increasing our general health and well-being.

## Supplementary Information

Additional file 1. The coding structure for the core category.

## Data Availability

The data used to support the findings of this study are available from the corresponding author on reasonable request.

## Abbreviations

GT: Grounded theory
RCT: Randomised controlled trial
FG: Focus groups
HIT: High-intensity training
