# Supplementary material for: Negotiating a physically active life in tune with ageing: a grounded theory study of older persons’ experiences of participating in high-intensity interval training
Source: BMC Geriatr. 2025 Jan 4;25:11. doi: 10.1186/s12877-024-05635-5 (PMC11699672; doi:10.1186/s12877-024-05635-5)
Supplement: Supplementary file 1 — Additional file 1. The coding structure for the core category. [file 12877_2024_5635_MOESM1_ESM.docx]

**Additional file 1.** Outlining the coding structure for the core category ***Negotiating a physically active life in tune with ageing.***

| **Focused codes** | **Sub-categories** | **Categories** |
| --- | --- | --- |
| Enjoying the short and intensive exercise | **Vitalised by challenging oneself** | **Grit in the moment and overall life** |
| Individual settings facilitate exercising for anyone |  |  |
| Gaining new insights about exercise and movement |  |  |
| Finding physical improvements with HIT | **Harvesting the benefits of exercising** |  |
| Acknowledging various psychological gains from training |  |  |
| Increasing physical exercise in general |  |  |
| Doing more everyday activities |  |  |
| Increasing intensity in activities |  |  |
| Feeling unsure about effects of the training |  |  |
| Exercising is greatly aided by the structure of the group | **Acknowledging the merits of exercising in a group** | **Empowered by the training group** |
| Valuing the social connectedness in the group |  |  |
| Willing to exert oneself more in a group |  |  |
| Being acknowledged and supported by coaches |  |  |
| Adjusting training intensity to suit one’s own needs | **Preparing for continued exercise without the group** |  |
| Feeling insecure about one’s capability to continue training |  |  |
| Finding other modalities |  |  |
| Finding support from others |  |  |
| Looking for similar activities |  |  |
| Acknowledging limitations for staying active | **Relating to one’s own active ageing in social contexts** | **Navigating one’s physically active self** |
| Recognising the uncertainty of ageing |  |  |
| Finding it hard to identify as an older person |  |  |
| Becoming more aware of one’s own mortality |  |  |
| Avoiding being dependent on others |  |  |
| Finding it hard to identify with people in other generations |  |  |
| Finding new priorities in life |  |  |
| Being approached as if one is fragile | **Trying to stay active in the transformation to pensioner** |  |
| Requiring own drive to stay active |  |  |
| Preconceived ideas about oneself | **Finding own drive to exercise** | **Committing to exercise for duty and pleasure** |
| Feeling safer to exercise after health checkup |  |  |
| Safeguarding against deterioration |  |  |
| Breaking a sedentary lifestyle |  |  |
| Needing something to do |  |  |
| Exercising for pleasure and joy |  |  |
| Appreciating support from relatives, friends and society | **Acknowledging social support and availability to exercise** |  |
| Environment and seasonal changes affect exercising |  |  |
| Having opportunity to exercise |  |  |
